# Effects of Smartphone Use on Sleep and Mental Health in Young Adults: Going Beyond Self-Report

**DOI:** 10.1155/da/3249012

**Published:** 2025-11-21

**Authors:** Elena Bild, Kalina R. Rossa, Shannon L. Edmed, Cassandra L. Pattinson, Dwayne L. Mann, Karen A. Sullivan, Paul M. Salmon, Sylistah Gadam, Arvind Gnani Srinivasan, Simon S. Smith

**Affiliations:** ^1^School of Psychology, The University of Queensland, Brisbane, Queensland, Australia; ^2^Child Health Research Centre, The University of Queensland, South Brisbane, Queensland, Australia; ^3^The Australian Research Council Centre of Excellence for Children and Families Over the Life Course, The University of Queensland, Brisbane, Queensland, Australia; ^4^The Australian Research Council Centre of Excellence for the Digital Child, The University of Queensland, Brisbane, Queensland, Australia; ^5^School of Psychology and Counselling, Queensland University of Technology, Brisbane, Queensland, Australia; ^6^The Centre for Human Factors and Systems Science, University of the Sunshine Coast, Sunshine Coast, Queensland, Australia; ^7^Institute for Social Science Research, The University of Queensland, Brisbane, Queensland, Australia

**Keywords:** anxiety, depression, mental health, sleep, smartphone, young adult

## Abstract

**Background:**

Poor sleep has been associated with mental health concerns such as anxiety and depression. Prior evidence suggests that smartphone use may be a factor in poor sleep and mental ill-health in young adults, though most studies have relied on self-reported measures of smartphone use and sleep, which can be unreliable. This study used objective and subjective measures to examine the relationship between time spent using smartphones, sleep duration, quality, regularity, and symptoms of depression and anxiety in a sample of self-selected poor sleepers.

**Methods:**

Participants (*N* = 99; 70.7% female) wore an actigraph for 2 weeks to assess their habitual nightly sleep duration and regularity. Their average daily smartphone screen use was collected over 1 week with a smartphone application. Standardized questionnaires were used to assess sleep quality and mental health symptoms.

**Results:**

No statistically significant associations were found between objective smartphone screen use and any sleep or mental health variables. Sleep disturbance, sleep-related daytime impairment, anxiety, and depression were positively correlated. However, regression models identified that only sleep-related daytime impairment explained unique variance in anxiety and depression when adjusted for sleep disturbance and duration, gender, age, and screen time.

**Limitations:**

Specific timing of smartphone screen use (e.g., evening use) and/or application content were not collected as part of this study.

**Conclusions:**

These results conflict with prior evidence demonstrating negative relationships between self-reported smartphone screen use, sleep, and mental health. Further research incorporating objective measurement of smartphone screen use, focusing on critical periods for sleep, may provide a more nuanced picture of this relationship. Results also demonstrate the differing roles of night-time sleep disturbance and daytime sleep-related impairment in mental health.

**Trial Registration:** Australian New Zealand Clinical Trials Registry number: ACTRN12621000132842

**Summary**

• Objective 24-h habitual smartphone screen use is not related to mental health or sleep.
• Disparity with prior findings warrants further exploration using objective measures of smartphone screen use and sleep.
• Daytime sleep-related impairment appears to be a unique predictor of mental health.

## 1. Introduction

It is well established that sleep is essential for healthy physical, emotional, and cognitive functioning. Yet many young adults sleep less than the recommended 7–9 h per night for optimal health [1]—and up to 60% of young adults report sleep problems, including difficulties with falling asleep and waking frequently [2].

Poor sleep may be a significant contributor to mental health challenges in young adults. Both depression and anxiety symptomatology are associated with delayed bedtime, more time spent in bed awake, perceived poorer sleep quality and/or quantity, and irregular sleep schedules [3–5]. Though the relationship between sleep and mental health is complex and bidirectional [6], evidence suggests that enhancing young adult's sleep can improve mental health [7, 8]. One factor that has been found to impact sleep in young adults is smartphone use [9].

Many studies have observed negative associations between screen time, particularly smartphone use, and sleep. For example, a systematic review of digital media use in late adolescence and young adulthood reported relationships between increased digital media use (including smartphone use) and delayed bedtime, shorter sleep duration, increased daytime tiredness, worsened sleep quality (although some findings were mixed), and early awakenings [9]. Though research capturing the dimension of sleep regularity is still emerging, increased smartphone use has been associated with inconsistent sleep habits [10] and sleep timing variability in young people [11].

Several reasons have been posited to explain the positive associations between smartphone use and sleep problems. First, smartphone use can displace time that the individual would otherwise spend sleeping [12]. Second, smartphone use can involve consuming content, which increases psychological and physiological arousal, making it more difficult to switch off and fall asleep [12]. A third factor is that smartphone devices emit light, particularly short-wavelength (blue) light. This light exposure can disrupt the body's production of melatonin, a hormone influencing the sleep–wake cycle, leading to increased evening alertness [12]. Finally, using smartphones in bed may also establish an association between being in bed and wakefulness, rather than sleep [13].

Self-reported smartphone use is known to be associated with increased mental health symptoms [14–17]. There are many proposed reasons for this association: First, smartphone use may result in fewer face-to-face interactions with friends and family [18]. Time on social media may result in increased attention problems, antisocial behavior, and body dissatisfaction [18]. Screen time may displace time otherwise spent completing academic or work tasks, or engaging in health-promoting activities such as physical activity and sleep [18]. Finally, the type of screen-based activity may be a factor. For example, the prevalence of depression associated with the use of social media, online gaming, and online video viewing is higher than that associated with watching television [19].

The existing literature offers substantial evidence supporting the associations between screen time (including smartphones), mental health (e.g., depression and anxiety), and sleep problems [9, 20, 21], likely through a range of biopsychosocial interactions. Increased smartphone use is associated with poorer sleep, as indicated, and shorter sleep duration and poor sleep quality are associated with mental health, potentially due to changes in neural substrates and underlying mechanisms associated with the regulation and control of emotion, behavior, and stress as a result of sleep loss [22, 23]. This pathway may, wholly or in part, explain the relationship between screen use (specifically, smartphones) and increased mental health symptoms (e.g., [18]).

Substantial heterogeneity of the measurement tools and approaches has been noted in systematic reviews on the relationship between digital media use, sleep, and mental health in young people [21, 24]. Alonzo et al. [24] noted that studies were limited by the use of self-report methods of sleep duration/quality and screen time/technology use. Smartphone use is typically measured by asking participants how long they spend on their phones or other devices, or by using a questionnaire to assess phone addiction. Smartphone screen use is subject to recall bias, with participants often underestimating time spent on devices [25], and participants who use their phone more may underestimate their screen use by a higher amount [26]. Although there have been a small number of recent studies using objectively measured smartphone use to examine associations with sleep [27–30], these studies are heterogeneous in their measurement approach, most used only self-reported sleep measures, and yielded inconsistent findings. Subjective sleep reports (capturing quality, duration, and timing) are subject to a negativity bias and are often underestimated and ill-defined in comparison to objective sleep measures, particularly in individuals experiencing depression and/or insomnia symptoms (both of which are associated with increased screen use) [31, 32]. For example, Andersen et al. [27] found clear associations between self-reported nighttime phone use, high perceived stress, and severe depression symptoms (i.e., higher odds of mental health concerns with self-reported greater nighttime phone use). However, the association was much less clear when using an objective measurement of nighttime phone use, suggesting that objective measures of smartphone use may produce weaker effects than self-reports.

This study aimed to investigate the independent effects of smartphone screen use and sleep-related factors (sleep duration, sleep disturbance, and sleep-related daytime impairment) on mental health symptoms (anxiety and depression). We hypothesized that smartphone screen use would be associated with changes in both anxiety and depression symptoms, and that the inclusion of sleep duration, sleep disturbance, and sleep-related daytime impairment would account for additional variance in anxiety and depression outcomes. This study extends prior research through the use of objective measures of sleep duration and smartphone screen use, alongside self-reported sleep quality. Furthermore, we also included an objective measure of sleep regularity in exploratory correlational analyses. The use of objective measurements may clarify the relationship between these factors and help to build the evidence base for directing interventions and treatment guidelines for individuals with problematic smartphone use and/or mental health issues.

## 2. Methods

### 2.1. Participants and Design

This study used a cross-sectional correlational design utilizing baseline data from a placebo-controlled, randomized control trial “Reducing Crash Risk for Young Drivers” [33]. A sample of 99 young adults aged 18–24 years (*M* = 20.6, SD = 1.9, 70.7% female gender) was recruited via advertisement in university and other higher education environments, social media, and a participant registry. Full participant eligibility criteria are detailed elsewhere [34], but study-relevant criteria are reported in Table 1.

### 2.2. Materials

#### 2.2.1. Patient-Reported Outcomes Measurement Information System (PROMIS) Measures

This study used measures from the PROMIS created by the National Institutes of Health (NIH). These measures were devised to increase standardization in patient-reported outcomes, are typically normed to allow comparisons across domains, have very strong psychometric properties, and are increasingly included as standard measures in research internationally [35].

##### 2.2.1.1. Sleep Disturbance

The PROMIS Sleep Disturbance Short-Form 8a measures self-reported sleep quality, depth, and restoration associated with sleep, including difficulty with getting to or staying asleep and perceived adequacy and satisfaction with sleep over the past 7 days [36]. The 8-Iitem scale includes questions such as “My sleep was refreshing” (reverse scored) and “I had difficulty falling asleep.”

##### 2.2.1.2. Sleep-Related Impairment

The PROMIS Sleep-Related Impairment Short-Form 8a measures self-reported perceptions of alertness, tiredness, and sleepiness during the day and perceived functional impairments associated with sleep over the past 7 days [37]. The 8-item scale includes questions such as “I had a hard time getting things done because I was sleepy” and “I felt irritable because of poor sleep.”

Both measures use the same 5-point Likert scale for rating items (1 = not at all to 5 = very much). The total score for each scale is the sum of item ratings, with higher scores indicating worse sleep disturbance or sleep-related impairment, respectively. Both measures have demonstrated convergent validity with other commonly used measures of sleep quality and are able to differentiate between participants with and without diagnosed sleep disorders [38].

#### 2.2.2. Mental Health

Poor mental health symptoms were measured using the PROMIS Short-Form v1.0 Anxiety 8a and Depression 8b scales.

##### 2.2.2.1. Anxiety

The PROMIS Anxiety scale measures self-reported fear, anxious misery, hyperarousal, and somatic arousal symptoms over the past 7 days [39]. The 8-item scale includes questions such as “I felt fearful” and “My worries overwhelmed me.”

##### 2.2.2.2. Depression

The PROMIS Depression scale measures self-reported negative mood, views of self, social cognition, and decreased positive affect and engagement over the past 7 days [40]. The 8-item scale includes questions such as “I felt worthless” and “I felt that I had nothing to look forward to.”

Both measures use the same 5-point Likert scale for rating items (1 = never to 5 = always), with higher scores indicating greater symptoms of mental ill-health. The PROMIS anxiety and depression short forms have excellent internal consistency (Cronbach's *α* = 0.93 and *α* = 0.95, respectively [41]).

### 2.3. PROMIS Scoring

PROMIS measures were scored using the free *HealthMeasures* online scoring system (https://www.assessmentcenter.net/ac_scoringservice). The PROMIS measures convert to T-scores with a mean of 50 and SD of 10. The cut points for the PROMIS sleep and mental health measures are <55 = within normal limits; 55–60 = mild; 60–70 = moderate; and 70–80 = severe.

#### 2.3.1. Objective Habitual Sleep Duration: Actigraphy

Small wrist-worn tri-axial accelerometers (actigraphs; GeneActiv Original, Activinsights Limited, Cambridge, UK) were used to estimate objective habitual sleep duration. The actigraph records raw movement signals together with ambient light and temperature (°C) over extended periods of time (up to 60 days). Actigraphy is the gold standard of objective sleep measurement in naturalistic studies [42–44]. It is analogous to polysomnography (PSG) as a valid and reliable measure of sleep variables, including sleep onset and wake times [45]. GeneActiv accelerometers have been validated against PSG in adult [46] and child [47] populations. The actigraphs were fully charged and configured to record at a sampling frequency of 10 Hz. Participants were asked to wear the watch on their nondominant wrist continuously for a minimum period of 2 weeks, ensuring an equal proportion of weekend and weekday recordings across participants. Habitual average nightly sleep duration (hours:minutes) was calculated from the available sleep periods collected during this measurement period, with periods of wake after sleep onset (WASO) removed.

#### 2.3.2. Sleep Regularity

The sleep regularity index (SRI), an objective measure of sleep regularity, was derived from actigraphy data using published methods [48]. The SRI measures how consistent a person's sleep–wake pattern is each day, based on a two-state sleep/wake classification. The metric calculates the probability of an individual being in the same state (i.e., either awake or asleep) at any two time points 24-h apart, averaged across the study, and scaled to range from 0 (*random*) to 100 (*perfectly regular* [48]).

#### 2.3.3. Average Daily Habitual Smartphone Screen Use

Objective 24-h habitual smartphone screen use was measured using participants' smartphones. When participants attended the lab, we asked them to access the digital wellbeing app or screen time function on their Android/iPhone device to record their daily total smartphone screen use (in hours:minutes) for the past 7 days. The screen time function is a measure of active screen time (e.g., scrolling on social media, using maps), not passive use (e.g., listening to music). The durations were averaged to determine average daily habitual smartphone use.

### 2.4. Procedure

Following recruitment, participants were screened for eligibility and reported demographic information. Participants then received an actigraph to wear for 2 weeks while living “life-as-usual.” When participants returned the actigraph to the lab, they reported their smartphone screen use data from their phones under the supervision of a research assistant and completed the questionnaires assessing sleep and mental health (see protocol [34] for full measures and procedure).

### 2.5. Data Processing: Actigraphy

Sleep periods were determined by algorithm-based procedures with human oversight. In brief, raw data were downloaded via GeneActiv software (version 3.3) and processed via two discrete, yet converging methods. Raw acceleration data (movement in *x*, *y*, and *z* axes) were processed using the R-Script “GGIR” (version 2.5 [49]) to derive sleep timing estimates. Raw data (*x*, *y*, *z*, light, and temperature) were also imported into Matlab (R2022b, Natick, Massachusetts, United States) and processed to determine Euclidean Norm Minus One (ENMO [50]) and arm angle/activity [51]. Processed data were then down-sampled using median values to 15-s epochs.

Review of the actigraphy-derived sleep timing was aided by the visualization of GGIR sleep onset and offset estimates overlayed upon signals from MATLAB-processed data. In this manner, a trained scorer (EB) then used contextual information (temperature and light) in addition to activity data to adjust the GGIR-based sleep timing estimates. For example, initial sleep timing may have overestimated sleep when periods of no-wear appear near contiguous with a period of sleep; however, this can be isolated by identifying abrupt changes in the corresponding temperature signal. The timing of sleep bouts, including sleep onset (bedtime) and offset (wake-time), was determined from the reviewed sleep periods per individual. WASO was then determined by automatically identifying segments of sustained activity within sleep, and was subtracted from total sleep opportunity (sleep onset to wake time) to provide sleep duration for each sleep bout. Each participants' habitual sleep duration was then calculated as the average sleep duration (hours:minutes) from all available valid sleep bouts (minimum of 3 sleep bouts, akin to 3 nights of data). To ensure scoring accuracy, 10% of the scored data were independently checked by a second researcher (KRR). The average number of days analyzed was 12.45 (SD = 2.642, range = 3–14).

### 2.6. Data Analysis

Data exploration and statistical analyses of cleaned and processed data were conducted using IBM Statistical Package for the Social Sciences (SPSS) Version 29.0. Missing data were observed for average daily habitual screen use (*n* = 13 missing), actigraphy-derived sleep duration (*n* = 5 missing), and PROMIS depression (*n* = 1 missing). The five individuals with missing data from actigraphy were excluded, leaving 94 participants with sleep duration data suitable for analysis. The remaining missing data were handled using pairwise deletion. We further explored whether individuals with missing data on the screen use variable differed systematically from those with complete data across key demographic variables. We observed that missingness was more common among males (62%; *n* = 8) than females (38%; *n* = 5). Average daily smartphone screen use and actigraphy derived sleep duration (both collected in hours:minutes) were converted to decimal hours to aide statistical modelling. All variables were normally distributed, apart from the SRI, which was negatively skewed (Shapiro–Wilk test *p* < 0.001). The variable was transformed using a reflect and square root to produce normality [52]. However, no differences were noted between raw and transformed data; as such, we report findings based on the raw data.

First, Pearson's *r* correlations were conducted to examine associations between continuous variables. Next, two hierarchical (sequential) multiple linear regressions were conducted on the outcome variables of the PROMIS depression and anxiety scales. Prior to conducting analyses, we confirmed that key hierarchical regression assumptions were met, including linearity, independence of errors, homoscedasticity, normality of residuals, and absence of multicollinearity. We selected the “enter” method to add variables based on theoretical temporal precedence [52]. In the first step, we entered our covariates (age and gender). In the second step, we entered the average daily habitual smartphone time use. In the third step, we entered sleep duration, sleep-related impairment, and sleep disturbance. This approach allowed us to explore whether the sleep-related variables explained additional variance in anxiety/depression symptoms beyond demographic and behavioral (smartphone use) factors.

## 3. Results

### 3.1. Sample Characteristics

#### 3.1.1. Sleep

Our sample had an average sleep duration over a 2-week period of 7 h 21 min (SD = 44 min), which is within recommended sleep guidelines for their age [1]. The mean *T*-score for sleep-related impairment was 60.05 (SD = 6.92). Subjectively, 21.2% of participants reported sleep-related impairment within normal limits, 25.3% reported mild sleep-related impairment, 48.3% reported moderate sleep-related impairment, and 5.1% indicated severe sleep-related impairment. For sleep disturbance ratings (*T*-score *M* = 52.12, SD = 5.09), 67.7% fell in the normal range, 24.2% reported mild sleep disturbance, and 8.1% reported moderate sleep disturbance.

#### 3.1.2. Mental Health

Of our participants' anxiety (*T*-score *M* = 57.47, SD = 8.45), 43.4% fell in the normal range for anxiety, 18.2% were in the mild range, 29.3% reported moderate anxiety levels, and 9.1% fell in the severe range. For depression (*T*-score *M* = 55.02, SD = 7.36), 54.5% were in the normal range, 17.2% reported mild levels, 27.3% reported moderate depression levels, and 1% fell in the severe range. See Table 2 for descriptive statistics.

### 3.2. Correlations

Pearson's *r* correlation coefficients indicated that habitual smartphone screen use was not associated with any sleep duration (*r* = −0.065) or mental health variables (anxiety *r* = −0.035, depression *r* = 0.015). Increased sleep-related impairment was positively associated with increased sleep disturbance (*r* = 0.368, *p* < 0.01), and increased anxiety (*r* = 0.512, *p* < 0.01) and depression symptoms (*r* = 0.453, *p* < 0.01). Further, increased sleep disturbance was associated with longer average sleep duration (*r* = 0.310, *p* < 0.01) and increased anxiety (*r* = 0.260, *p* < 0.01) and depression symptoms (*r* = 0.346, *p* < 0.01). Increased anxiety symptoms were associated with increased depression symptoms (*r* = 0.774, *p* < 0.01). See Table 3 for the correlation matrix for smartphone screen use, sleep, and mental health variables. See Supporting Information for correlation plots.

Exploratory correlational analyses examining associations between sleep regularity, smartphone screen use, other sleep and mental health variables indicated no relationship between regularity of sleep and any other variable (Table 3).

### 3.3. Effect of Smartphone Screen Use and Sleep on Depression Symptoms

The effects of smartphone screen use and sleep variables on depression were examined using hierarchical multiple regression. The covariates of age and gender were entered into the model in Step 1, explaining 0.1% of the variance in depression. An additional 0.1% variance was explained by adding average smartphone screen use into the model at Step 2, *F*(3,73) = 0.57, *p*=0.982. After the entry of sleep-related impairment, sleep disturbance, and average sleep duration at Step 3, the model explained a total of 25.5% variance in depression, *F*(6,70) = 4.003, *p*=0.002. The sleep variables significantly explained additional variance in depression after covariates and smartphone screen use were controlled for (*R*^2^_change_ = 0.253, *p* < 0.001). In the final model, only sleep-related impairment was statistically significant (*β* = 0.405, *p*=0.001). Semi-partial correlations showed that, while sleep disturbance and sleep-related impairment had significant zero-order correlations with depression, only sleep-related impairment explained unique variance in depression (Table 4).

### 3.4. Effect of Smartphone Screen Use and Sleep on Anxiety Symptoms

The effects of smartphone screen use and sleep variables on anxiety were examined using hierarchical multiple regression. The covariates of age and gender were entered into the model in Step 1, explaining 4.2% of the variance in anxiety. An additional 2.5% variance was explained by adding average smartphone screen use into the model at Step 2, *F*(3,73) = 1.762, *p*=0.162. After the entry of average sleep duration, sleep-related impairment, and sleep disturbance at Step 3, the model explained a total of 38.1% variance in anxiety, *F*(6,70) = 7.181, *p* < 0.001. The sleep variables significantly explained additional variance in anxiety after covariates were controlled for (*R*^2^_change_ = 0.313, *p* < 0.001). In the final model, only sleep-related impairment was statistically significant (*β* = 0.572, *p* < 0.001). Semi-partial correlations showed that, while sleep disturbance and sleep-related impairment had significant zero-order correlations with anxiety, only sleep-related impairment explained unique variance in anxiety (Table 5).

## 4. Discussion

This study used a cross-sectional design to examine the extent to which objectively measured smartphone screen use, and a combination of objective and subjective sleep measures (duration, quality, daytime sleep-related impairment and regularity), predict mental health symptoms (anxiety and depression) in young adults. Contrary to expectations, we found that time spent using a smartphone screen was not associated with any sleep or mental health variable.

Our results are inconsistent with previous literature in this area, which suggests a relationship between smartphone use and sleep quality or mental health [21, 24]. One potential explanation for the inconsistency between our results and previous studies is heterogeneity in measurement. Most prior research has assessed screen time by asking participants to self-report the amount of time spent on devices doing a particular activity (e.g., viewing television or gaming/other computer-related tasks [18, 53, 54]). Not only are these retrospective reports often subject to recall bias [26], but the type of use captured in these studies is relatively discrete in comparison to that which may have been captured through our study using a continuous objective measure of habitual smartphone screen use (24 h/day across a week). The gamut of uses that are afforded by the smartphone today (e.g., social media and messaging, emails, navigation, gaming, general internet browsing, shopping, forums, and educational tasks) is significantly different to that captured via self-report with existing measures.

Although an early review found similar effects on sleep across different types of devices [12], most of the studies noted in this review were assessing self-reported device use and/or sleep. The relationship between mental health, smartphone screen use, and sleep may become nonsignificant when employing objective measures. For example, a study conducted by Rod and colleagues [55] demonstrated a nonsignificant association between mental health and using a smartphone during sleep when employing an objective measure of screen time, although a significant negative association between objective screen time and *self-reported* sleep duration *was* found. Similarly, Mac Cárthaigh et al. [29] found negligible yet significant negative associations between objective problematic smartphone use and *self-reported* sleep quality; however, no significant associations were found between objective problematic smartphone use and *objective* sleep duration as measured via wearable sleep trackers. Discrepancies between objective measurement and subjective perception of a range of sleep domains are common in healthy sleepers and may become more pronounced in individuals experiencing symptoms of mental ill-health or existing sleep disorders such as insomnia [31, 56, 57]. As our study is one of the first to use objective measures of *both* smartphone use and sleep duration, there are very few studies available for direct comparison. The inconsistency between our findings and that of previous literature highlights the need for further *objective* measurement of sleep *and* smartphone use.

Furthermore, the timing of screen use during the day may be important for sleep or mental health-related factors. For example, previous studies that have assessed smartphone use and sleep or mental health have found significant negative associations between *bedtime use* and sleep. A systematic review found that high use around bedtime was most strongly associated with depressive symptoms, potentially due to content from the news or social media increasing arousal, anxiety, and distress [24]. Moreover, high screen use before bedtime may decrease melatonin production [58] and shift circadian timing [12], with implications for mental health. As smartphone screen use was expressed in daily totals in our study, we cannot distinguish between daytime and evening use. Evening use in particular shares mechanistic links with disrupted and short sleep due to the proximity of the device to specialized sleep and circadian modulating cells in the retina [59]. Some of the participants in our study with high smartphone screen use may have been using their phones during the day for work/learning or for specific functions such as GPS-guided navigation, and our measure did not enable us to distinguish this use from participants using their phones in the evenings prior to bedtime. To address this, some studies asked participants to report their screen use not including use for work or school, and also found associations with sleep (e.g., [60, 61]) and mental health variables (e.g., [53, 54]). On balance, although other studies have found effects with an all-day measure of screen use (e.g., [11, 62]), our global measure of smartphone screen use may have been insensitive to variations in the extent of use around bedtime, contributing to the discrepancy in results. This highlights the need for future objective smartphone screen use measurements to be sensitive to timing and content.

### 4.1. Sleep and Mental Health

We found that greater sleep disturbance and greater sleep-related impairment had significant bivariate associations with poorer mental health. However, when we entered both into our regression models, only sleep-related impairment explained unique variance in anxiety and depression. Given that sleep-related impairment and mental health symptoms likely have a bidirectional relationship, the implied directionality of our analysis should be interpreted with acknowledgement of this complexity. Additionally, objectively measured sleep duration was not associated with either mental health variable, and exploratory analyses found no significant relationship between objective sleep regularity and mental health.

Our study considered both sleep disturbance and sleep-related impairment, two dimensions that are often combined within global sleep scales (e.g., the Pittsburgh Sleep Quality Index; PSQI [63], and the Insomnia Severity Index (ISI [64])). However, these dimensions are likely to be associated with mental health in different ways. The use of two separate scales enabled us to understand these differing effects more clearly. Greater sleep disturbance and sleep-related impairment were related to greater symptoms of anxiety and depression. However, only sleep-related impairment explained unique variance in anxiety and depression. The shared variance between these predictors can be interpreted as the single underlying construct of sleep quality. However, the additional unique variance shared between the mental health variables and daytime sleep-related impairment may be due to two potential explanations. First, hyperarousal, a stress response resulting in increased sympathetic nervous system activation [65], can cause daytime fatigue and exhaustion. An individual with an insomnia-like phenotype may attribute these symptoms to poor sleep, even if their sleep was objectively and subjectively satisfactory [66]. Our sample reported greater sleep-related impairments than expected, given their subjective sleep experience. Increased anxiety and depression symptoms can result in higher levels of hyperarousal due to more frequent activation of the fight or flight system and higher cortisol levels [65]. Consequently, increased hyperarousal may account for the unique variance that sleep-related impairment explained in anxiety and depression. Alternatively, daytime sleepiness and fatigue in young people can be caused by “social jetlag,” where societal schedules (e.g., early work and school start times) are at odds with body clocks that reflect natural preferences for later bedtimes [67, 68], leading to sleep curtailment and irregular sleep timing. Research has identified that variability in sleep timing can result in negative mood [69, 70], mood disorders [71], and poor wellbeing [68] in adolescents. However, we found no association between our measures of anxiety, depression, and smartphone screen use with objective sleep regularity (Table 3). This may be due to methodological differences in quantifying sleep timing variability (see [48] for review), and in the differences inherent between adolescents and young adults in exercising personal autonomy in response to educational, vocational, and social demands on time. Our participants appeared to have generally good sleep regularity, especially for a younger cohort, which may explain the lack of significant association found between regularity and sleep-related impairment in this sample. Further research is needed to explore the relationship between regularity, associated daytime impairment, and mental health in young adults.

### 4.2. Limitations and Future Directions

A potential limitation of our study was the broadness of our measure of smartphone use. As discussed earlier, we recorded a singular number of smartphone usage over a 24-h period, so we may have missed a more nuanced picture of the relationships between the *timing* of smartphone use, mental health, and sleep. Other studies that have objectively measured smartphone use have gathered this data by downloading a specific app onto participants' phones [72] or providing a new smartphone to participants [55]; however, we chose to try using a built-in function already available on participants phones. Using a purpose-built application to capture greater objective detail on the timing of use, and on the content being consumed (e.g., application choices), would be beneficial for future research.

Additionally, our inclusion criteria for the broader project may have impacted the results of our study. First, the entry criteria entailed the sample self-identifying as poor sleepers, endorsing the inclusion criteria of sleeping less than 7 h per night on average, and less than 6 h on 2 or more nights per week. However, the majority (70.2%) had an average sleep duration of the recommended 7 h or more [1], and most participants fell in the normal (67.7%) or mild (24.2%) range of the sleep disturbance scale. Our sample population differs from participants in most other studies cited, where average sleep durations were shorter (e.g. [11, 73, 74]) and subjective sleep quality was poorer (e.g. [72, 75]). As most of our sample could be regarded as good sleepers, this may have been protective against any potential negative effects of smartphone screen use and mental health disturbances. Second, we excluded participants taking psychotropic medication, which may have impacted the variability in our mental health measures. Further research that specifically captures a representative cohort of young adults is warranted to understand these associations more clearly than what is available to date.

A further limitation of our study is that we were unable to match objective sleep durations with daily screen use. Our study employed a retrospective cross-sectional design. Objective measures of “habitual sleep duration” were collected over a 2-week period, while “habitual smartphone use” was assessed over a 7-day period immediately prior to the laboratory visit. Notably, these assessment periods did not always overlap. For some participants, actigraphy data were obtained 2–3 weeks prior to the laboratory visit. To infer causal relationships between screen use and mental health, future studies could examine these data prospectively and match smartphone usage to same-day sleep duration collected via actigraphy. Additionally, there is likely a direct mechanistic pathway linking sleep-related factors (duration, disturbance, and daytime impairment) and mental health symptoms. Future research could test these causal mediation pathways using longitudinal designs or formal mediation models to better understand the indirect effects of sleep-related factors on mental health outcomes.

Recruitment and data collection for this study were conducted during a period that included both the COVID-19 pandemic and the 2022 Brisbane floods. These events introduced logistical challenges, particularly in relation to participant recruitment, which was constrained by a fixed end date due to funding requirements. As such, our sample size was limited. Additionally, as the sample consisted primarily of employed university students, the generalisability of our findings may be impacted.

## 5. Conclusions

In conclusion, our study provides important new insights into the relationship between smartphone screen use, sleep, and mental health by using objective measures, contrasting with most prior research that relied on self-reported data. Given the ubiquitous use of smartphones in daily life, particularly among young adults, understanding how screen use impacts sleep and mental health is critical for public health. Future research should continue to explore these relationships using objective measures to help inform interventions and treatment approaches for issues involving smartphone use, mental health, and sleep disturbances.

## Figures and Tables

**Table 1:** Participant eligibility and exclusion criteria.

Eligibility criteria	Exclusion criteria
• Every day access to a smartphone device • Self-reported sleep of less than 7 h per night on average, and less than 6 h on at least 2 nights per week • Aged 18–24	• Overnight shift worker (i.e., any shifts falling between 12 a.m. and 6 a.m.) • Travel across >3 time zones within the last 3 months or intention to travel within the first 3 months of the study • Current use of over-the-counter substances with psychoactive properties • Pregnant or planning a pregnancy in the following 10-month period • Self-reported medical diagnosis of a sleep disorder and/or eating disorder • Use of medically prescribed stimulants, antidepressants, antianxiety, antipsychotic, mood stabilizing medications, sleep medications, or appetite suppressants • Sole carer of a child • Regular smoker

**Table 2:** Descriptive characteristics for smartphone screen use, sleep, and mental health variables.

	*N*	Mean	SD	Min	Max
*Objective measures*					
Smartphone screen use (decimal hours)	86	5.53	1.93	1.98	10.33
Average sleep duration (decimal hours)	91	7.35	0.74	5.27	8.93
Sleep regularity index	93	74.96	9.52	41.27	98.86
*Subjective measures*					
PROMIS-SR	99	60.05	6.92	40.60	73.90
PROMIS-SD	99	52.12	5.09	40.80	63.80
PROMIS Anxiety	99	57.47	8.45	37.10	78.10
PROMIS Depression	99	55.02	7.36	37.10	71.10

*Note: T*-scores are reported for all PROMIS measures.

Abbreviations: PROMIS-SD, PROMIS sleep disturbance; PROMIS-SRI, PROMIS sleep-related impairment.

**Table 3:** Correlation matrix for smartphone screen use, sleep, and mental health variables.

	Smartphone screen use	Average sleep duration	Sleep regularity index	PROMIS-SRI	PROMIS-SD	PROMIS anxiety	PROMIS depression
Smartphone screen use	—						
Average sleep duration	−0.065	—					
Sleep regularity index	−0.106	0.088	—				
PROMIS-SRI	0.061	−0.072	−0.030	—			
PROMIS-SD	0.059	0.310*⁣*^*∗∗*^	0.098	0.368*⁣*^*∗∗*^	—		
PROMIS anxiety	−0.035	0.044	0.001	0.512*⁣*^*∗∗*^	0.260*⁣*^*∗∗*^	—	
PROMIS depression	0.015	0.087	−0.004	0.453*⁣*^*∗∗*^	0.346*⁣*^*∗∗*^	0.774*⁣*^*∗∗*^	—

Abbreviations: PROMIS-SD, PROMIS sleep disturbance; PROMIS-SRI, PROMIS sleep-related impairment.

*⁣* ^*∗*^ *p* < 0.05.

*⁣* ^*∗∗*^ *p* < 0.01.

**Table 4:** Regression predicting depression scores from covariates, smartphone screen use, and sleep variables.

Variables		*B*	*β*	*t*	*p*
Step 1	Gender	0.427	0.026	0.222	0.825
	Age	0.102	0.027	0.230	0.819
Step 2	Gender	0.572	0.032	0.269	0.789
	Age	0.070	0.019	0.153	0.879
	Smartphone screen use	−0.123	−0.036	−0.288	0.774
Step 3	Gender	−0.831	−0.051	−0.467	0.642
	Age	0.137	0.036	0.335	0.738
	Smartphone screen use	−0.143	−0.041	−0.378	0.706
	PROMIS-SRI	0.367	0.405	3.427	0.001
	PROMIS-SD	0.248	0.180	1.457	0.150
	Average sleep duration	0.217	0.024	0.218	0.828

*Note: B*, unstandardized coefficients.

Abbreviations: PROMIS-SD, PROMIS sleep disturbance; PROMIS-SRI, PROMIS sleep-related impairment.

**Table 5:** Regression predicting anxiety scores from covariates, smartphone time use, and sleep variables.

Variables		*B*	*β*	*t*	*p*
Step 1	Gender	2.764	0.141	1.229	0.223
	Age	−0.579	−0.129	−1.117	0.267
Step 2	Gender	3.331	0.170	1.467	0.147
	Age	−0.759	−0.168	−1.430	0.157
	Smartphone screen use	−0.699	−0.168	−1.413	0.162
Step 3	Gender	2.144	0.110	1.104	0.274
	Age	−0.603	−0.134	−1.355	0.180
	Smartphone screen use	−0.741	−0.178	−1.796	0.077
	PROMIS-SRI	0.622	0.572	5.314	<0.001
	PROMIS-SD	−0.020	−0.012	−0.106	0.916
	Average sleep duration	0.652	0.061	0.601	0.550

*Note: B*, unstandardized coefficients.

Abbreviations: PROMIS-SD, PROMIS sleep disturbance; PROMIS-SRI, PROMIS sleep-related impairment.

## Data Availability

The data that support the findings of this study are available from the corresponding author upon reasonable request.

## Supporting Information

Supporting Information 1 Supplement A contains scatterplots of the correlations reported in Table 3.
