# Supplementary figures and images for: Effects of Smartphone Use on Sleep and Mental Health in Young Adults: Going Beyond Self-Report
Source: Depress Anxiety. 2025 Nov 21;2025:3249012. doi: 10.1155/da/3249012 (PMC12662671; doi:10.1155/da/3249012)

**Supplement A.** Correlation plots.


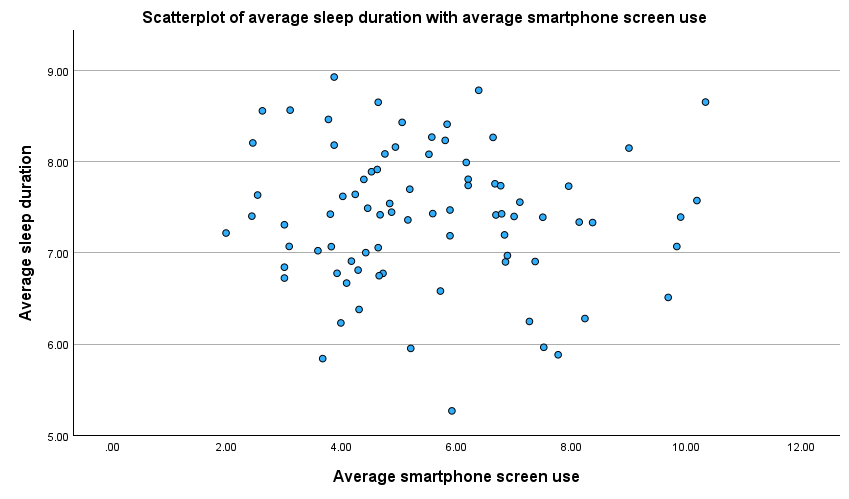

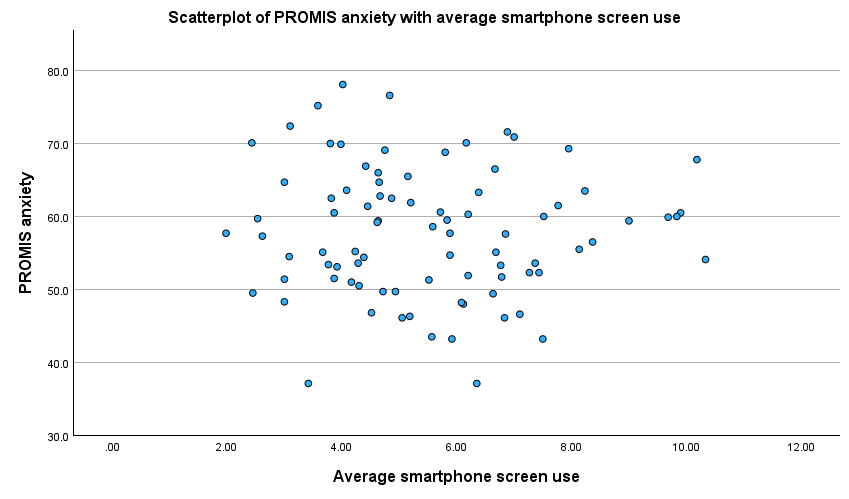


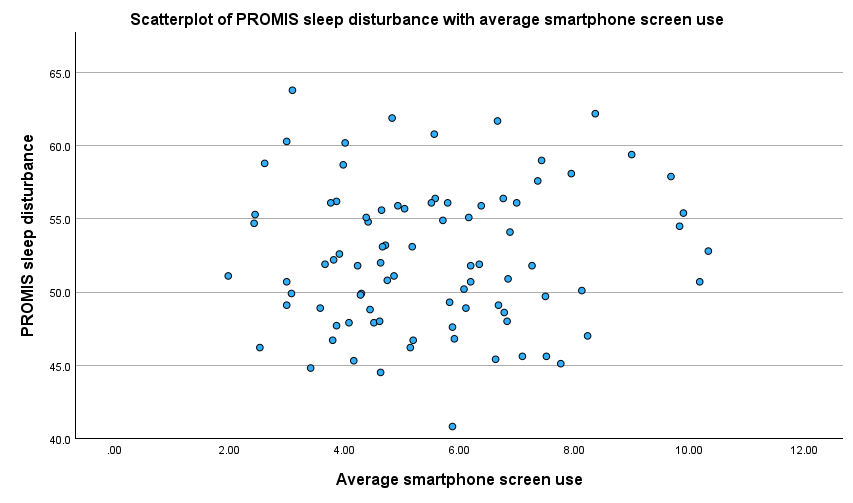

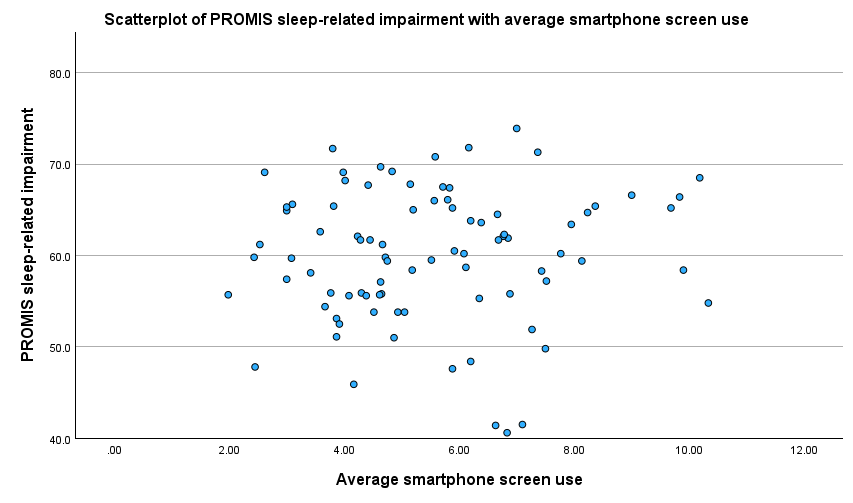


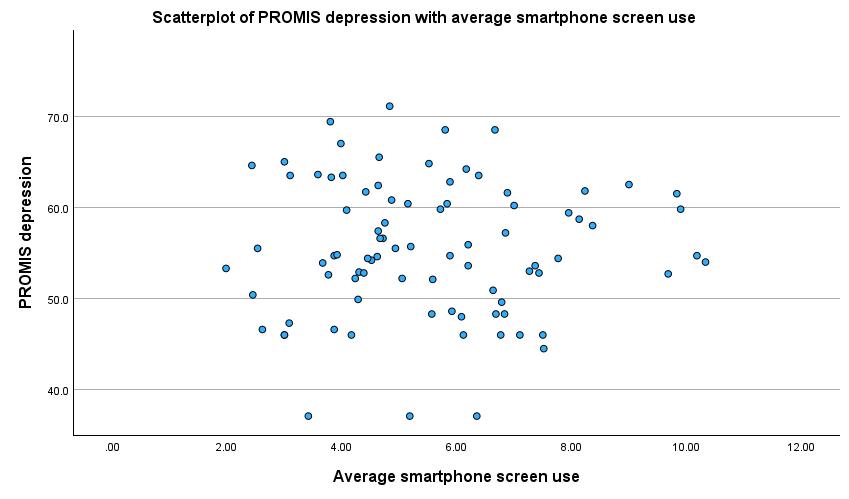

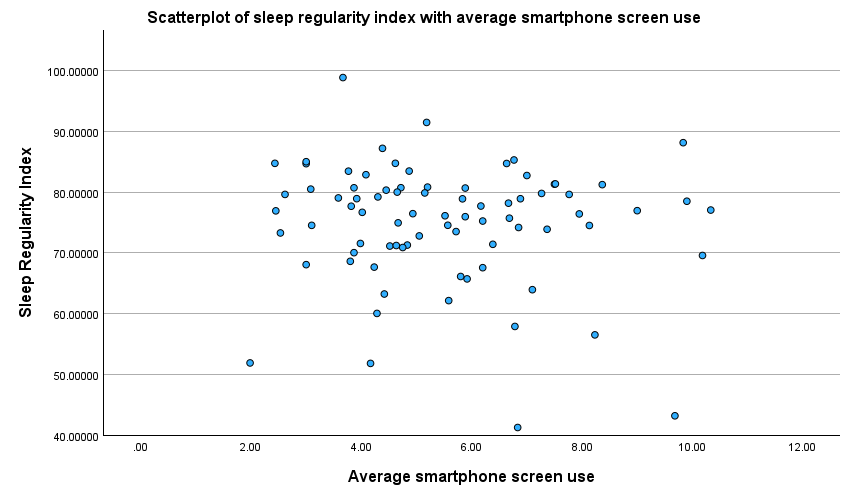


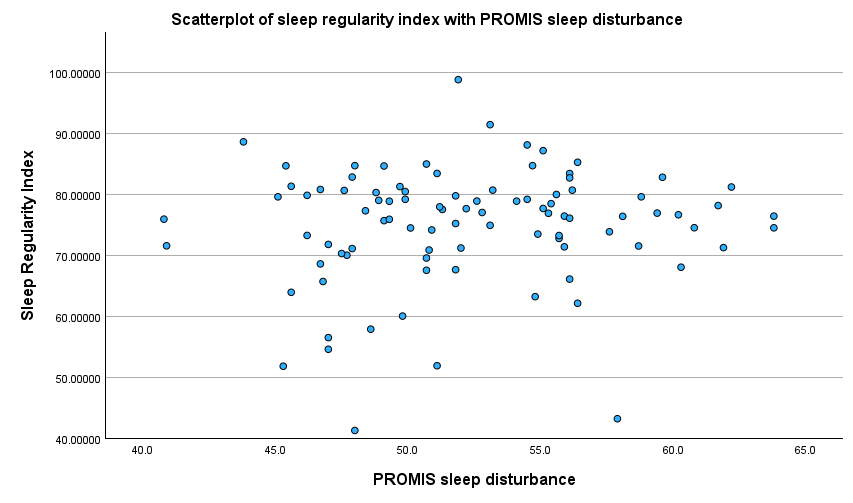

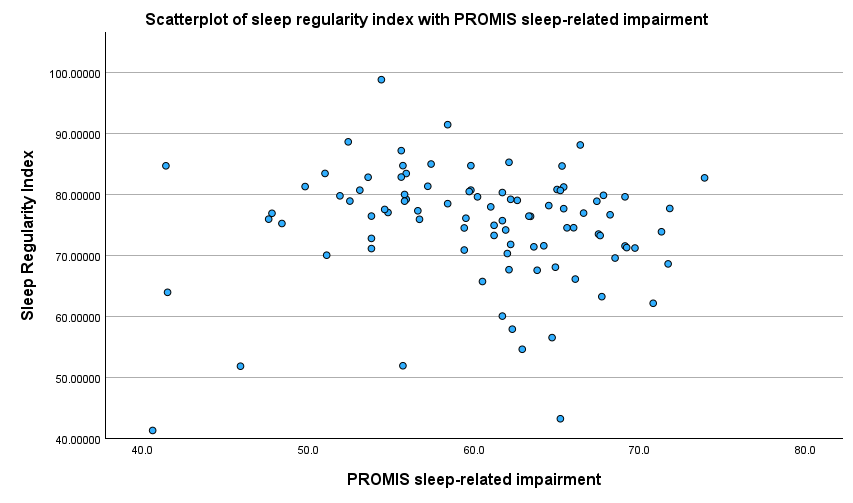


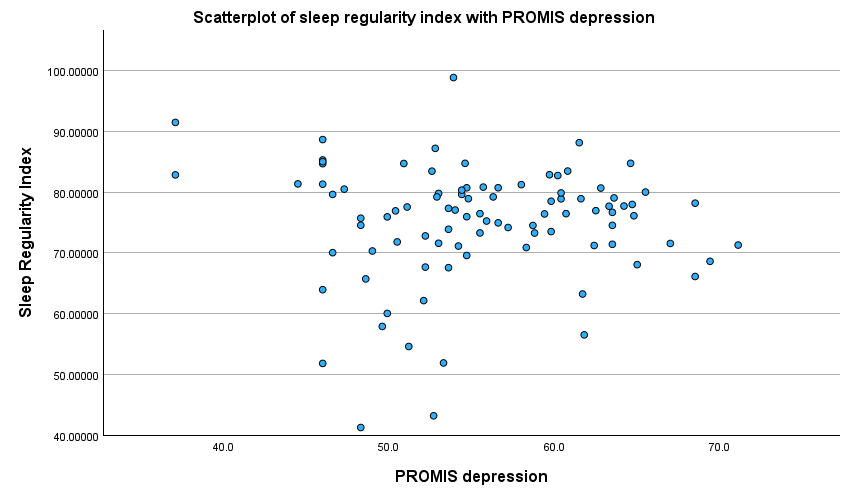

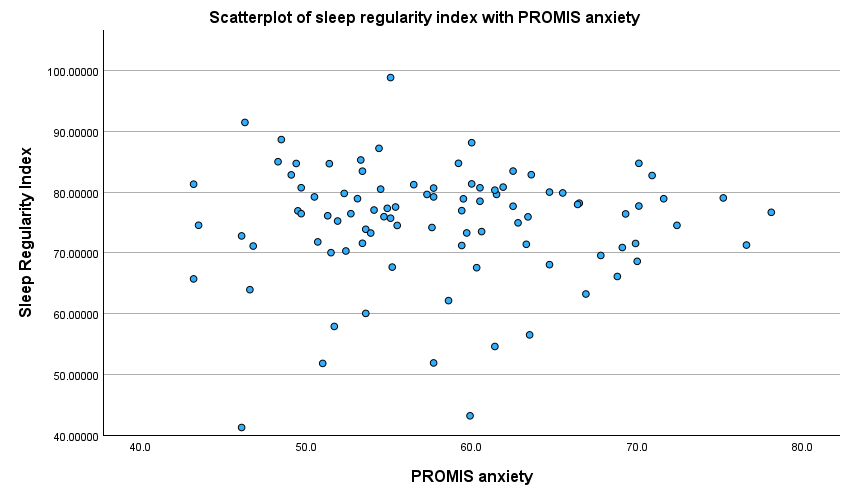


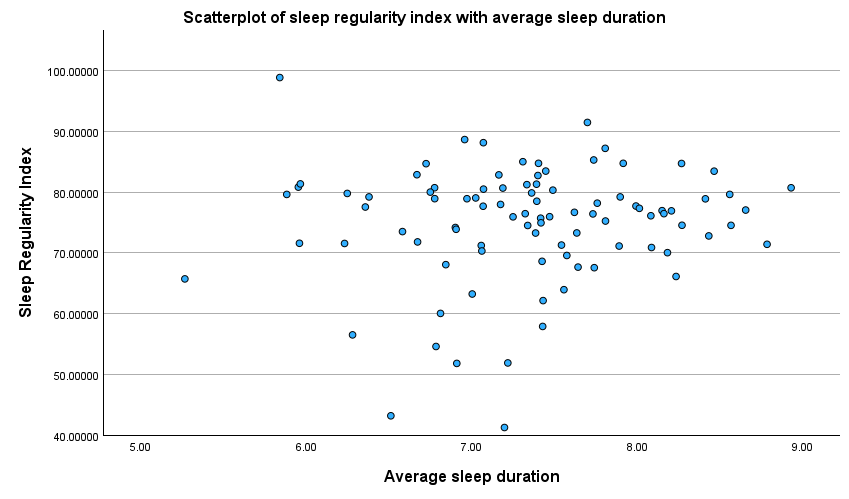

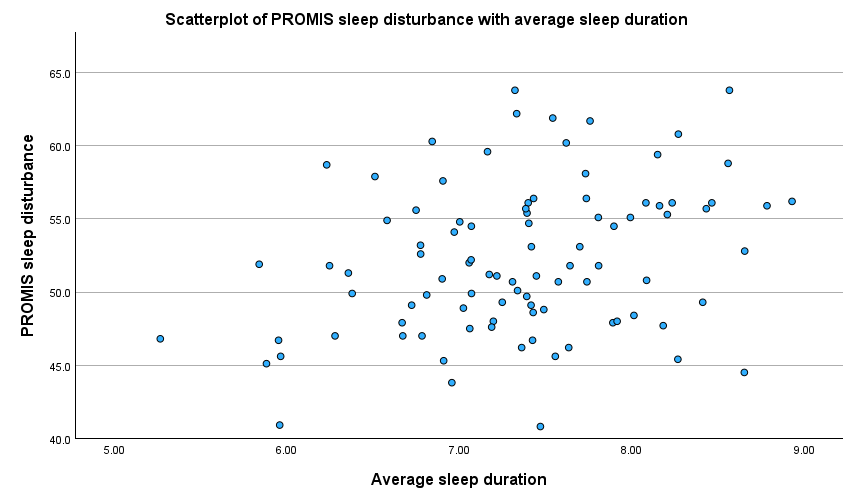


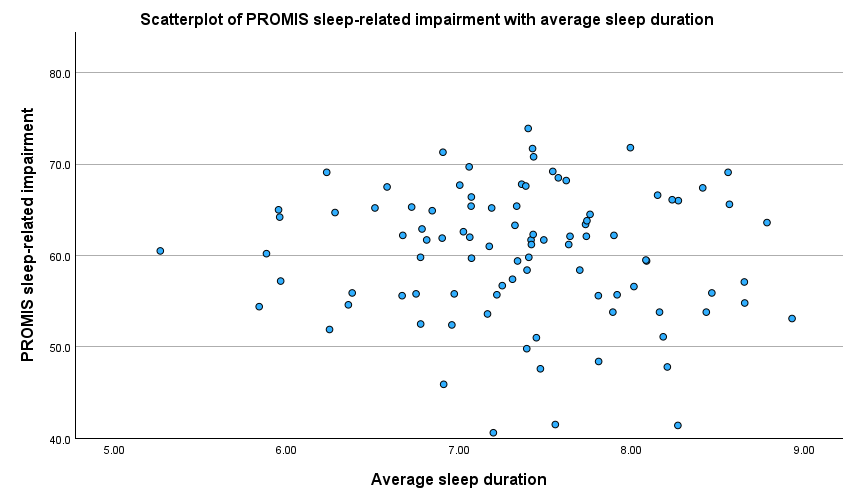

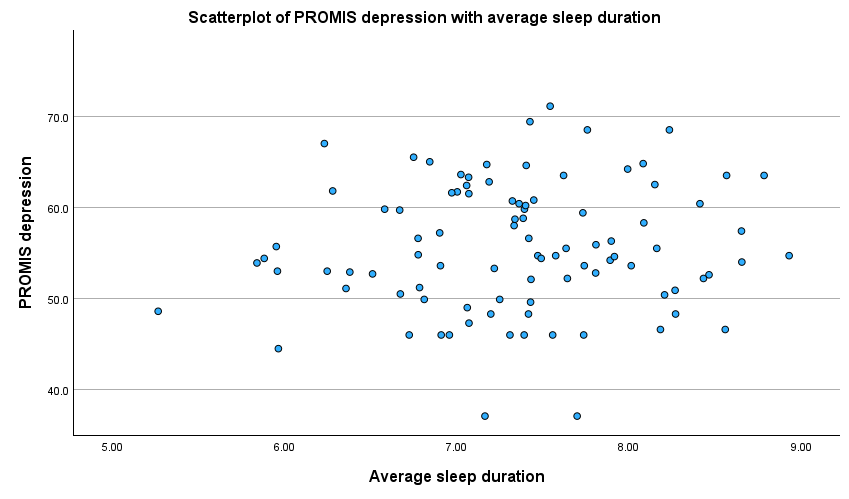


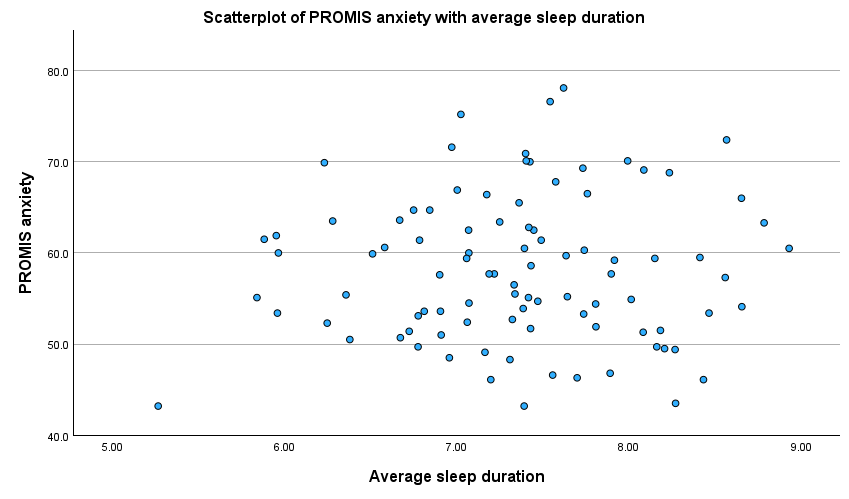

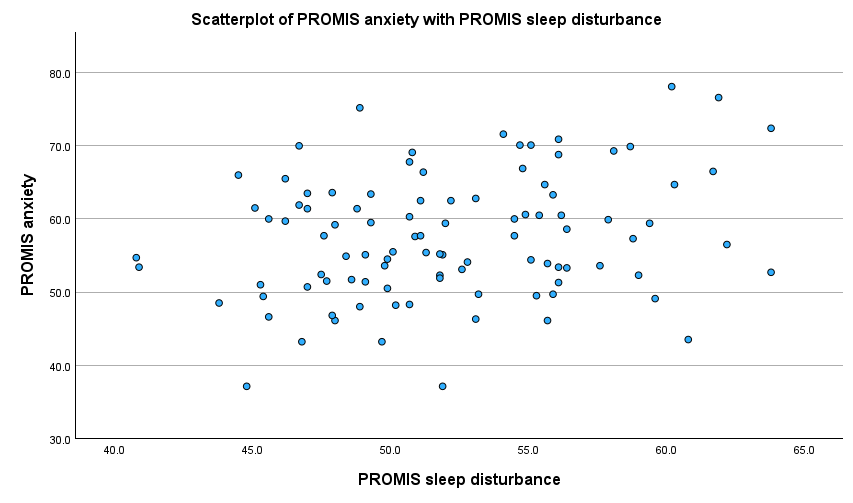


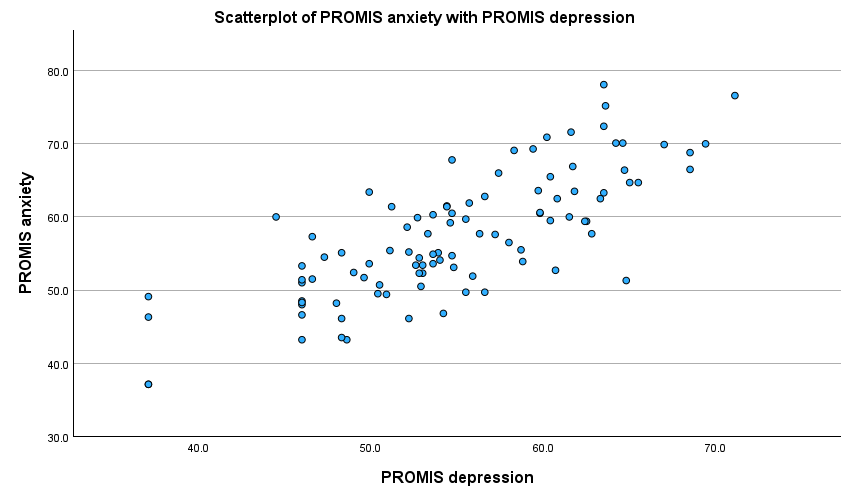

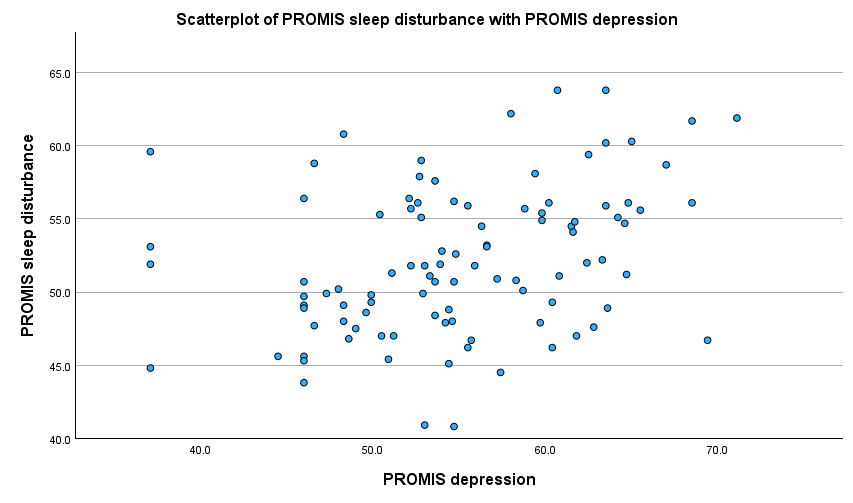


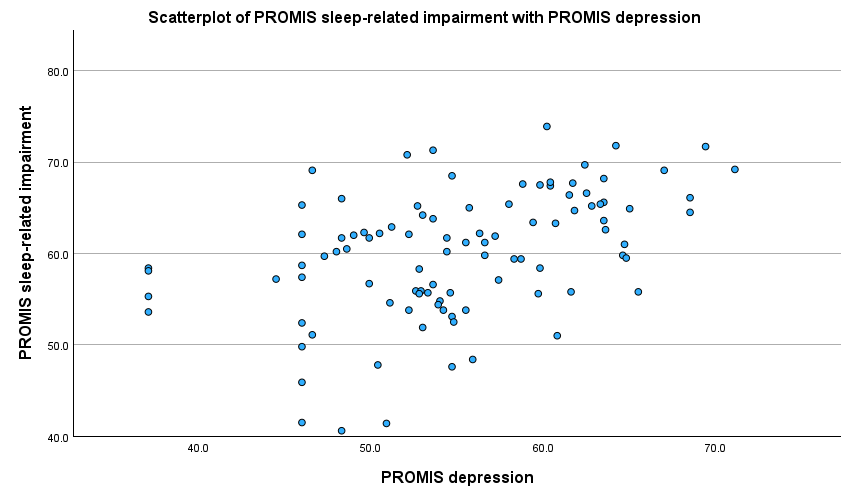

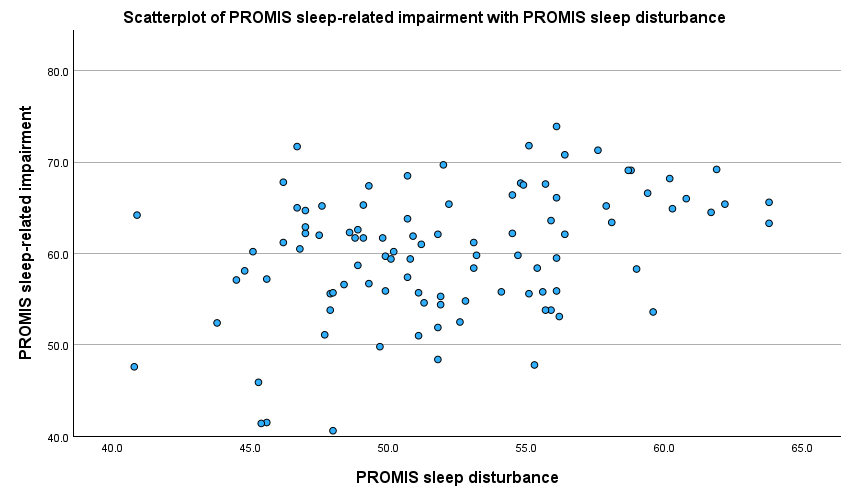


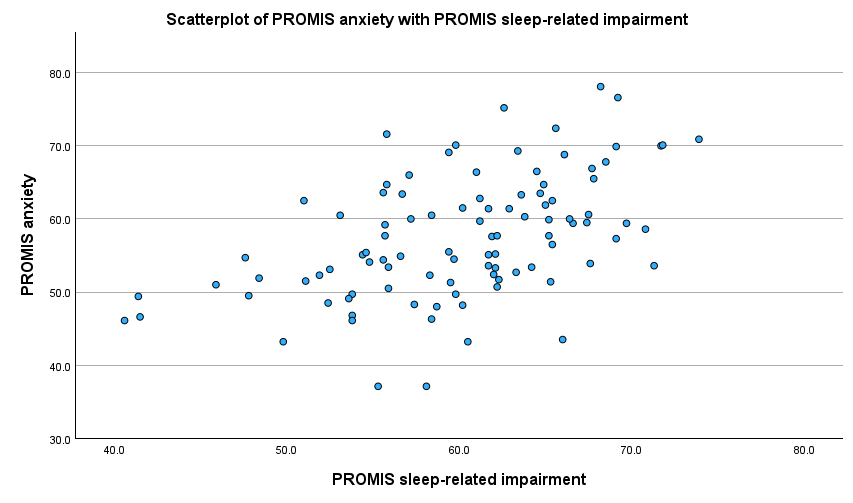

Supplement: Supporting Information 1 — Supplement A contains scatterplots of the correlations reported in Table 3. [file 3249012.f1.docx]
